# Regulation of RIC-3 and of nAChR expression

**DOI:** 10.18632/oncotarget.13925

**Published:** 2016-12-12

**Authors:** Yael Ben-David, Millet Treinin

**Affiliations:** ^1^ Department of Medical Neurobiology, Hadassah Medical School-Hebrew University, Jerusalem, Israel

**Keywords:** RIC-3, nicotinic acetylcholine receptors, alternative splicing, cholinergic anti-inflammatory pathway, Neuroscience

The nicotinic acetylcholine receptors (nAChRs) form a large and diverse family of pentameric acetylcholine-gated ion channels which are expressed widely and have diverse roles. The best known nAChR, α1β1δε or α1β1δγ, is expressed in skeletal muscle and mediates muscle excitation by motor neurons. In neurons nAChRs have both excitatory and modulatory roles and in non-excitable cells they affect many processes including suppression of pro-inflammatory cytokine release from immune cells [1]. Moreover, CNS-expressed nAChRs have been implicated in several neurodegenerative diseases including Parkinson’s disease, dementia with Lewy bodies, and Alzheimer’s disease [2, 3].

Maturation of nAChRs is a complex and inefficient process requiring assistance from multiple cellular factors including RIC-3, a conserved endoplasmic reticulum-resident protein and nAChR-specific chaperone. RIC- 3 was first identified in the nematode *Caenorhabditis elegans* as a positive effector of nAChR maturation. Effects of mammalian RIC-3, however, were shown to be either positive or negative depending on the receptor and the experimental system [1]. The versatile effects of RIC-3 on co-expressed nAChRs and the diverse roles of nAChRs affected by it suggest that mechanisms regulating RIC-3’s function enable regulation of multiple processes involving nAChRs. One mechanism likely to affect RIC- 3 function is alternative splicing, as expression studies identified multiple alternatively spliced RIC-3 isoforms differentially expressed in multiple tissues [4].

To better understand effects of RIC-3 splicing on nAChR maturation we analyzed two conserved RIC-3 isoforms: one encoding for the full-length (FL) RIC-3 having two transmembrane domains followed by a coiled-coil domain, and the other encoding for the transmembrane domains only (TM). These two RIC-3 isoforms were examined for their effects on three major neuronal nAChRs (α3β4, α4β2 and α7) using electrophysiological analysis in *Xenopus laevis* oocytes. This analysis showed that the TM isoform lacking the coiled-coil domain is defective for either positive or negative effects of RIC-3 depending on the specific nAChR examined [5]. Specifically, FL amplified currents through α4β2 at low concentrations and inhibited currents at high concentrations; in contrast, FL inhibited currents through α7 at low or very high concentrations and amplified currents at a high concentration. TM did not amplify currents through α7 nAChR and did not inhibit currents through α4β2 nAChR at any concentration. FL and TM similarly amplified currents through α3β4 at low concentrations and inhibited currents at high concentrations [5].Together these results are consistent with previous results suggesting that RIC-3 interacts differently with different nAChRs [6].

Results from expression analysis show that the two RIC-3 isoforms express in the brain differentially and in immune cells RIC-3 expression and splicing are regulated by inflammatory signals. This regulation of RIC-3 expression and splicing is likely to have functional implications as electrophysiology results, summarized above, show either positive or negative effects of RIC- 3 on nAChR expression, depending on RIC-3 expression level, specific RIC-3 isoform, and specific nAChR [5].

Of special interest are effects of RIC-3 expression and splicing on the α7 nAChR. Effects of RIC-3 on this receptor are strongly dependent on RIC-3-to-receptor ratio and on the RIC-3 isoform [5]. The α7 nAChR is widely expressed, has many functions, and has been implicated in several neurodegenerative diseases [2, 7]. Among other functions, α7 nAChR mediates the cholinergic anti-inflammatory pathway in immune cells - a pathway enabling suppression of inflammation by the vagus nerve. Our results showing regulation of RIC-3 expression and splicing by inflammatory signals in immune cells suggest, therefore, that regulation of RIC-3 expression and splicing affect α7 nAChR functional expression and thereby inflammatory processes. Indeed, RIC-3 and the α7 nAChR have been implicated in neurodegenerative diseases in which neuroinflammation is known or is likely to have a role [7,8]. Future studies, therefore, should examine the role of RIC-3 and of mechanisms regulating its expression and splicing in neuroinflammatory diseases.
